# Supplementary figures and images for: Resurrection of 2′-5′-oligoadenylate synthetase 1 (OAS1) from the ancestor of modern horseshoe bats blocks SARS-CoV-2 replication
Source: PLoS Biol. 2023 Nov 28;21(11):e3002398. doi: 10.1371/journal.pbio.3002398 (PMC10683996; doi:10.1371/journal.pbio.3002398)

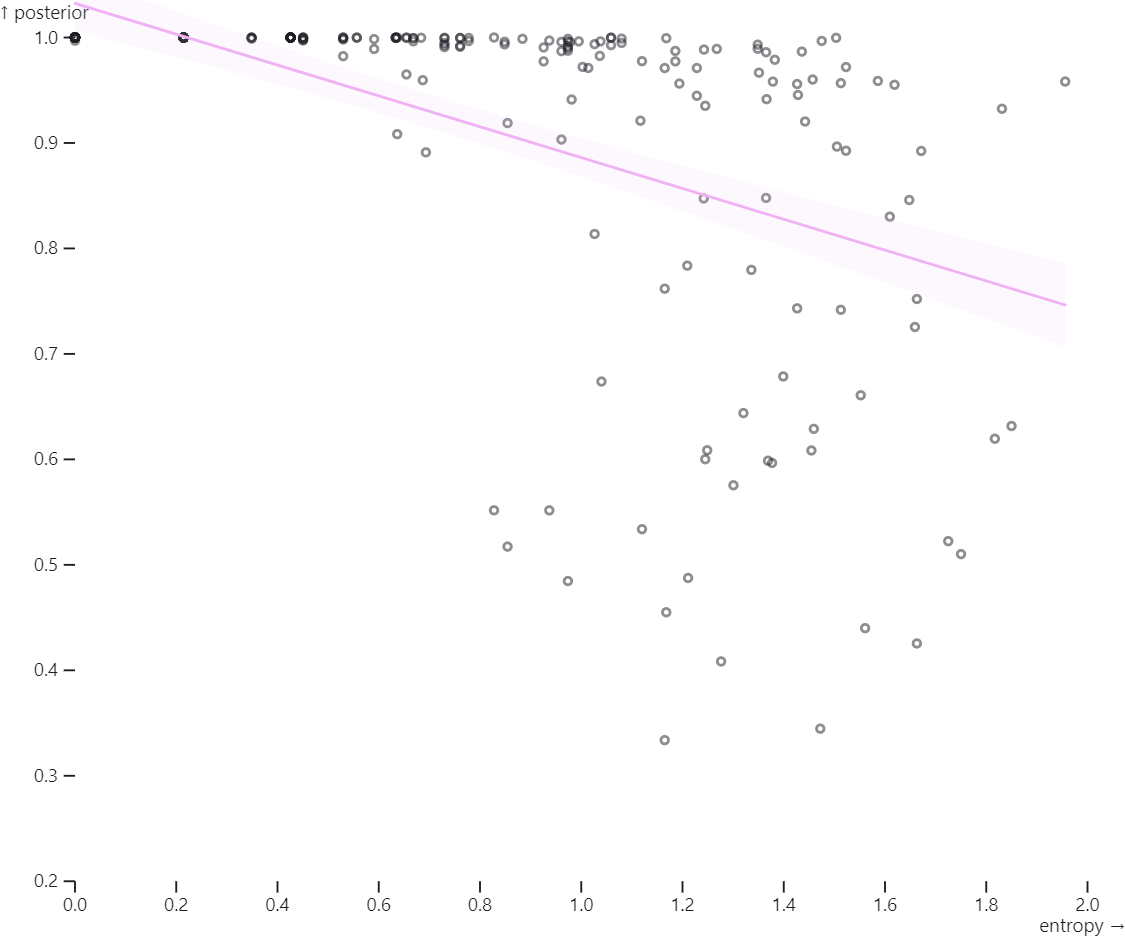

Supplement: S1 Fig — Dot plot showing Chiroptera OAS1 alignment entropy values and RhinoCA ancestral state reconstruction posterior values for each site in the alignment. Linear regression with confidence interval shading calculated with Observable HQ plots is presented. Sites with posterior values of 0 corresponding to multiple gaps have been removed from the plot. (TIF) [file pbio.3002398.s001.tif]

Figure 2A

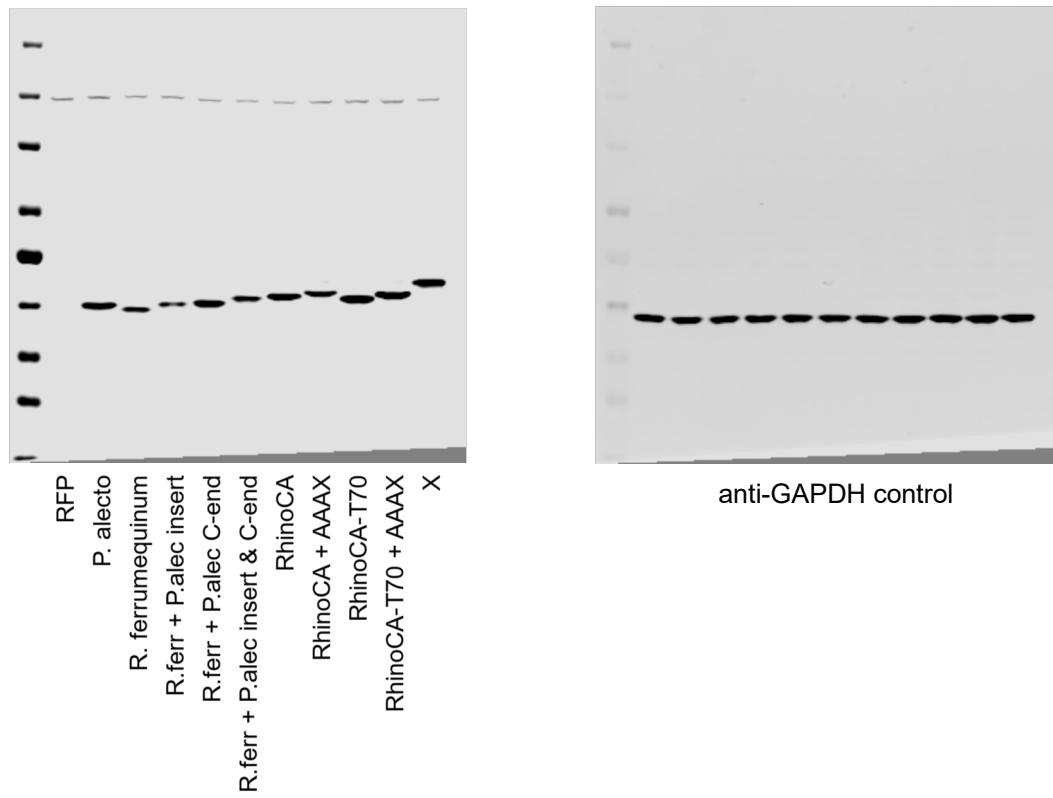

Figure 3C

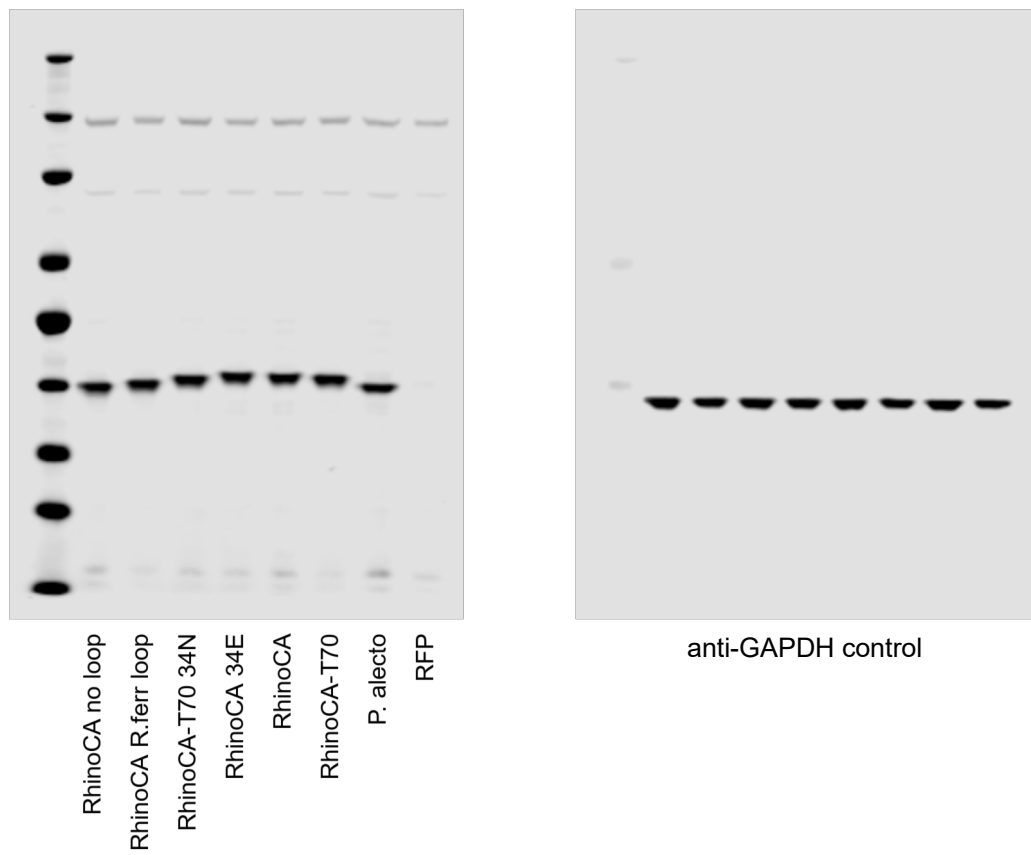

Supplement: S2 Data — Raw images for western blots supporting the experimental assays, corresponding to Figs 2A and 3C. (PDF) [file pbio.3002398.s004.pdf]
