# Supplementary material for: Resurrection of 2′-5′-oligoadenylate synthetase 1 (OAS1) from the ancestor of modern horseshoe bats blocks SARS-CoV-2 replication
Source: PLoS Biol. 2023 Nov 28;21(11):e3002398. doi: 10.1371/journal.pbio.3002398 (PMC10683996; doi:10.1371/journal.pbio.3002398)
Supplement: Attachment — Submitted filename: reviewer_response.docx [file pbio.3002398.s005.docx]

Response to reviewers

Reviewer #3: General
The authors previously reported that Rhinolophus bats' OAS1 lost its antiviral activity against SARS-CoV-2 due to the truncation of the C-terminal region, resulting in the loss of prenylation motif. In this study, the authors reconstructed the ancestral sequence of Rhinolophus bats' OAS1 and revealed that the OAS1 of Rhinolophus bats' ancestors had antiviral activity against SARS-CoV-2. Furthermore, they showed that not only the loss of prenylation motif but also the various mutations accumulated during the evolution of Rhinolophus bats could be involved in the inactivation of antiviral activity against SARS-CoV-2. However, they have not experimentally demonstrated which mutations, other than the prenylation motif, are involved in the inactivation of antiviral activity against SARS-CoV-2. The authors should address this point by experiments.

Major
Major Comment 1
The authors should clearly state the specific objective of this study in the last paragraph of the introduction. In the second-to-last paragraph of the introduction, the authors present a hypothesis regarding the evolutionary story of Rhinolophus bats' OAS1 and SARS-related CoVs. In the final paragraph, the authors state that they reconstructed the ancestral sequence of Rhinolophus bats' OAS1 and examined its properties in order to test this hypothesis. However, there seems to be a logical gap between the hypothesis and the strategy. The authors should define the specific objective and fill in the logical gap. In my understanding, the specific objective is to clarify how Rhinolophus bats' OAS1 lost its inhibitory activity against SARS-related CoVs. Is my understanding correct?

We thank the reviewer for this comment. Indeed, the data presented in the paper do not tackle the direct relevance of coronavirus PDE evolution to host OAS1 antiviral activity and the link between the two paragraphs is rather confusing. We have now rewritten the last paragraph of the introduction as follows:

*“In this study, we explore how the OAS1 protein lost its inhibitory activity against SARS-related coronaviruses at the base of the Rhinolophoidea superfamily and provide novel insights about the evolution of this protein in horseshoe bats. To do this, we phylogenetically reconstructed the ancestral OAS1 gene sequence likely present in the Rhinolophoidea common ancestor at the time of the LTR insertion.”*

We hope this clarifies the scope of the study.

Major Comment 2
The authors point out the importance of the variable indel region in the inhibitory activity against SARS-related CoVs. Additionally, in reconstructing the RhinoCA sequence for experimentation, they insert the variable indel region from P. alecto into the sequence estimated through ancestral sequence reconstruction. However, the importance of the variable indel region in the inhibitory activity against SARS-related CoVs has not been experimentally verified. The authors should examine the effect of the deletion on the inhibitory activity against SARS-related CoVs using RhinoCA as backbone sequence.

We agree with the reviewer that the relevance of the variable indel region to antiviral activity is an important point that was not addressed in the original submission. We have now experimentally tested the importance of the region by swapping it with the shorter *R. ferrumequinum* genotype or removing it altogether in the ancestrally reconstructed protein. Our new results suggest that in fact the variable indel region does not affect the protein’s antiviral activity.

We have appended a paragraph under the Results’ “Restored anti-SARS-CoV-2 activity in the ancestral OAS1 protein” section extensively describing these results, which are visually illustrated in the new Figure 3, panels C-E. The Discussion section has been modified accordingly based on the new findings.

Major Comment 3
In the discussion, the authors argue that the substitution in the 34th amino acid may be crucial for the inactivation of antiviral activity against SARS-related CoVs. This hypothesis should be experimentally tested. If the results from Major Comment 2 and 3 are obtained, the authors will be able to directly discuss how the ancestral OAS1 lost its antiviral activity against SARS-related CoVs through a series of events, which would increase the biological significance of this study.

We have now experimentally validated our original hypothesis about site 34 of the RhinoCA OAS1 protein by swapping the residues between RhinoCA and RhinoCA-T70. Our results show that, indeed, switching site 34 is sufficient for reversing the antiviral phenotype in the context of the RhinoCA OAS1 protein. We have now incorporated these results along with the *in silico* investigation of site 34 (previously presented in the Discussion and Figure S2) into the Results’ “Restored anti-SARS-CoV-2 activity in the ancestral OAS1 protein” section. The content of Figure S2 has been moved to the new Figure 3 (panels A and B) which also contains the relevant experimental results (panels C-E).

We hope that the new experimental results addressing the reviewer’s major comments 2 and 3 have increased the biological significance of our paper and expanded its scientific relevance with a better understanding of bat OAS1 biology. Exploring the exact, stepwise loss of the Rhinolophoidea OAS1 antiviral activity against SARSr-CoVs comprehensively would be very experimentally challenging, since RhinoCA OAS1 is about 60 residues different from the extant *R. ferrumequinum* OAS1, accumulated over 60 million years of evolution. We now explicitly clarify this in the Results’ “The Rhinolophoidea common ancestor OAS1 protein” section:

*“The RhinoCA and RhinoCA-T70 ancient OAS1 sequences differed from the extant R. ferrumequinum OAS1 protein at 57 and 61 protein sites respectively (excluding gaps), representing approximately 60 million years of evolution[10].”*

Nonetheless, the fact that we can demonstrably restore this protein’s antiviral function, lost so far in the evolution of bats, in addition to the novel insights in bat OAS1 function included in the manuscript after the reviewers’ recommendations should make this manuscript a significant scientific contribution to the field.

Minor
Minor Comment 1
In the main text, the term "RhinoCA" is used in two different contexts: one to represent the sequence estimated through ancestral sequence reconstruction, and the other to represent the actual sequence used in the experiments (which is the sequence estimated through ancestral reconstruction with the addition of P. alecto's variable indel region and C-terminal end sequence). It would be better to use two distinct words to distinguish these two contexts.

We apologise for the confusion, the RhinoCA OAS1 sequence should be the ancestral reconstruction with the *P. alecto* variable loop and C-terminal end (as shown in Figure 2C). We have now clarified this in the text as well:

*“We refer to this Rhinolophoidea common ancestor OAS1 protein as “RhinoCA” OAS1 (Figure 2C).”*

Given the reviewer’s comment we further wanted to clarify in the text that what we refer to as RhinoCA OAS1 is a best prediction of what the sequence of the extinct bat ancestor would have looked like. We have accordingly modified the abstract:

*“Herein, we used an ancestral state reconstruction approach to predict, and reconstitute in vitro, the most likely OAS1 protein sequence expressed by the Rhinolophoidea common ancestor prior to its prenylation loss (RhinoCA OAS1).”*

And amended the Results section accordingly:

*“Although it is impossible to know the true OAS1 protein sequence expressed by the Rhinolophoidea common ancestor, RhinoCA is the best prediction of the extinct protein sequence, given the available data.”*

Minor Comment 2
For Fig. 1A, the legend should be updated to indicate that the highlighted yellow section represents the CAAX box.

We have added this.

Minor Comment 3
In Fig. 3C, how about illustrating the 34th residue as well?

We now dedicate a main figure panel (Figure 3B) on the structural placement of site 34 on the RhinoCA OAS1 protein.

Reviewer #1: In this manuscript, Lytras and collaborators reveal that the loss of prenylation in horseshoe bat OAS1 causes this protein to lose its antiviral activity. However, OAS1 from a predicted common ancestor of Rhinolophoidea bats would retain prenylation and thus its antiviral activities. The authors also identified residues in Rhinolophus bat OAS1 under diversifying selection, which may be responsible for acquiring an alternative function in horseshoe bats, since the addition of prenylation signal did not rescue the antiviral activity of this protein. This is an exciting finding that is well supported by the data presented by the authors, and may help explain why rhinolophus bats are a major reservoir of coronaviruses. This reviewer has no concerns and recommends the manuscript for publication

We really appreciate the reviewer’s kind comments on our manuscript.

Reviewer #2: Oligoadenylate synthetase (OAS) proteins can sense viral double-stranded RNA (dsRNA), which activates RNase L by catalyzing the production of 2′-5′-oligoadenylate, resulting in cleavage of viral RNA and inhibition of viral infection. OAS1 has been identified as an interferon-stimulated gene (ISG) that strongly inhibits SARS-CoV-2 infection. In addition, this anti-SARS-CoV-2 activity requires prenylation of OAS1, which is determined by a CAAX box prenylation motif at the C-terminus of this host protein.

In this manuscript, Lytras et al. used bioinformatic approach to analyze OAS1 proteins in horseshow bats, where SARS-CoV-2 might be originated from. They found that OAS1 proteins lost the prenylation signal and did not exhibit any anti-SARS-CoV-2 activity. When the prenylation signal was repaired, these OAS1 proteins re-gained this antiviral activity. Although these findings are interesting, they simply re-confirm the important role of prenylation in OAS1 antiviral activity. In addition, although these results may explain how SARS-CoV-2 could evolve from the Rhinolophoidea, the significance of these findings is not sufficient to attract the broad audience of this journal.

We thank the reviewer for their comments. The manuscript has now been revised with additional insights about the function and biology of bat OAS1 proteins, prompted by reviewer 3’s suggestion, that we hope increase the overall significance of the manuscript and its appeal to a broader audience.
